# Opioid consumption frequency and its associations with potential life problems during opioid agonist treatment in individuals with prescription-type opioid use disorder: exploratory results from the OPTIMA Study

**DOI:** 10.1186/s12954-025-01157-4

**Published:** 2025-02-08

**Authors:** Anne Bouthillier, Gabriel Bastien, Christina McAnulty, Hamzah Bakouni, Bernard Le Foll, M. Eugenia Socias, Didier Jutras-Aswad

**Affiliations:** 1 Department of Psychiatry and Addictology, Faculty of Medicine, Université de Montréal, Montreal, QC Canada; 2 Research Centre, Centre Hospitalier de l’Université de Montréal (CRCHUM), Montreal, QC Canada; 3 Translational Addiction Research Laboratory, Campbell Family Mental Health Research Institute, Center for Addiction and Mental Health, Toronto, ON Canada; 4 Department of Pharmacology and Toxicology, Faculty of Medicine, University of Toronto, Toronto, ON Canada; 5 Department of Family and Community Medicine, Faculty of Medicine, University of Toronto, Toronto, ON Canada; 6 Department of Psychiatry, University of Toronto, Toronto, ON Canada; 7 Waypoint Research Institute, Waypoint Centre for Mental Health Care, Penetanguishene, ON Canada; 8 British Columbia Centre on Substance Use, Vancouver, BC Canada; 9 Department of Medicine, Faculty of Medicine, University of British Columbia, Vancouver, BC Canada

**Keywords:** Opioid use disorder, Harm reduction, Potential life problems, Opioid agonist treatment, Patient-centered outcomes, Opioid use frequency

## Abstract

**Background:**

Traditional treatment approaches for prescription-type opioid use disorder (POUD), centered on abstinence, have limitations and hinder the development of interventions that meet the needs of people with POUD. Reduction in use without complete abstinence presents a promising avenue for intervention enhancement, but supporting data is scarce regarding its translation into positive patient outcomes. This study explores whether reducing opioid use frequency (OUF) during opioid agonist treatment correlates with reduced potential life problems in individuals with POUD, including those using fentanyl.

**Methods:**

This study is an exploratory analysis of the OPTIMA trial, a pragmatic, open-label, randomized controlled study comparing the effectiveness of flexible take-home dosing of buprenorphine/naloxone and supervised methadone in reducing opioid use amongst individuals with POUD. OUF was assessed every two weeks for 24 weeks after treatment initiation using the Timeline Followback. Potential life problems were evaluated at baseline and study completion using the Addiction Severity Index Self-Report. The 114 participants who completed both baseline and end-of-study questionnaires were included. A repeated-measures generalized linear mixed model (GLMM) was used to evaluate the influence of OUF on potential life problems.

**Results:**

Reducing OUF was significantly associated with fewer problems related to medical status (*p* = 0.049), psychiatric status (*p* = 0.019), and alcohol problem severity (*p* = 0.001). The interaction was non-significant for employment (*p* = 0.264), family status (*p* = 0.352) and legal status (*p* = 0.050). Life improvements emerged with ≤ 21 days of opioid use per 28-day period.

**Conclusion:**

Findings underscore the significance of harm reduction goals focusing on opioid use reduction, which translated in improvements across many life domains.

**Trial registration:**

Study was registered with ClinicalTrials.gov (NCT03033732) prior to participant enrollment.

**Supplementary Information:**

The online version contains supplementary material available at 10.1186/s12954-025-01157-4.

## Background

The FDA’s approval of OxyContin in 1995 played a pivotal role in the onset of the opioid crisis in North America [1]. Alongside aggressive pharmaceutical marketing, overprescribing by healthcare providers, growing patient demand for pain relief, and the escalating availability of potent prescription-type opioids in the street market, including fentanyl and carfentanyl, have all contributed to the enduring opioid epidemic [2]. On a population-level, opioid use-related harms have been dreadful. In 2021, in the United States, fatalities resulting from drug overdoses exceeded six times the figure recorded in 1999 [3]. Opioids were implicated in over 75% of the approximately 107,000 drug overdose deaths reported that year [4]. The 2017 U.S. opioid epidemic incurred an estimated $1,021 billion economic burden, with $471 billion linked to opioid use disorder (OUD), primarily due to reduced quality of life [5]. Beyond these numbers, on an individual level, it has been shown that individuals with OUD, including prescription-type opioid use disorder (POUD), have worse mental and physical quality of life when compared to the general population [6, 7].

The negative impacts of prescription opioids non-therapeutic use have led to calls for comprehensive public health strategies – one key component being improving access to treatment of OUD with opioid agonist therapy (OAT) [8]. OAT that are recommended in American national guidelines for the treatment of OUD include methadone and buprenorphine [9]. In people with OUD, OAT have been shown to reduce overdoses [10, 11], to lower overall illicit opioid use [12] and crimes related to drugs [13], to limit the transmission of bloodborne diseases [14] and to increase adherence to HIV treatments [15, 16]. Methadone and buprenorphine/naloxone (BUP/NX) have also been associated with improvements in quality of life, as well as in physical and psychosocial function [17–20].

While OAT may exert positive effects on a variety of outcomes, the focus of substance use disorders treatment has historically been focused on achieving abstinence, meaning the complete cessation of drug consumption. This criterion has traditionally been a prerequisite for FDA approval of medications designed to address such conditions [21]. The emphasis on abstinence as the primary endpoint in clinical trials has been recognized as a significant obstacle in the development of new treatments [22], as it can be overly restrictive when considering the widespread nature of chronic and relapsing substance use disorders [23, 24].

Beyond the challenges of abstinence as a key treatment target for OUD, Alves et al. studied whether the outcome measurement of interventions did reflect the personal goals of people with substance use disorders, using a patient-centered perspective [25]. They found that outcome measures currently studied in substance use disorder treatments neglect several areas of concern for this population, including ability to care for oneself, outlook on life, feelings of guilt, dependence on others, and housing issues [25]. Such growing evidence of the benefits of patient-centered care and the call for harm reduction approaches for OUDs, notably in the context of the opioid epidemic, highlighted the need to identify more comprehensive endpoints that reflect decreased substance-related problems and broader improvements in well-being and functioning [26–28].

In light of the evolving understanding of the need for more holistic approaches in substance use disorder interventions, it is important to consider how these insights align with the response to the ongoing opioid epidemic. The FDA took a significant step in 2020 in response to this crisis by expanding its evaluation criteria for clinical trials assessing treatments for OUDs [29]. Among others, change in drug use pattern instead of total abstinence is now an accepted primary endpoint. However, the benefits of diminishing the frequency of use for individuals with OUD on potential life problems is not well established in the literature. A recent study has identified that the frequency of heroin use among methadone-maintained individuals was, among others, one of the factors associated with psychosocial functioning [7]. Another study found that self-reported frequency of heroin use was negatively associated with psychological quality of life following buprenorphine treatment [19]. Such associations have not been studied in the context of the use of increasingly potent prescription-type opioids (i.e., fentanyl, carfentanyl, etc.) which are now largely consumed in many jurisdictions around the globe and are associated with specific risks and overall worse outcomes [30–32].

The aim of this study is to determine whether a potential reduction in the opioid use frequency (OUF) during OAT is associated with a reduction in potential life problems in individuals with POUD.

## Methods

### Study design

This study is an exploratory analysis of the OPTIMA trial, a Canadian, multicentric, pragmatic, open-label, two arm parallel randomized controlled trial assessing the effectiveness of flexible take-home BUP/NX in comparison to supervised methadone for the treatment of POUD. The study was conducted in accordance with ethical standards outlined in the Declaration of Helsinki, the Tri-Council Policy Statement on Ethical Conduct for Research Involving Humans, and adhered to Good Clinical Practice guidelines and Health Canada division 5 guidelines. Approval was obtained from local research ethics committees, and the study was registered with ClinicalTrials.gov (NCT03033732) prior to participant enrollment. The methodology of the trial and its main outcomes have been previously published [20, 33].

### Participants

Individuals were aged 18–64, diagnosed with moderate to severe prescription-type opioid-use disorder based on DSM-5 criteria, seeking treatment and potentially benefiting from an OAT. Prescription-type opioids could be either licit or illicit, could have been prescribed or not, and could include fentanyl (synthetic fentanyl or misuse of prescription fentanyl). Exclusion criteria included having any unstable psychiatric or medical condition that would jeopardize safe study participation or compromise the ability to provide informed consent, having a pain condition requiring opioid treatment, heroin being the drug used most frequently in the last 30 days, treatment with OAT 30 days preceding screening, taking medication interacting with the trial medication, having history of severe adverse reaction to the study medication, having a pending legal case that might prevent study completion, pregnancy, breastfeeding or women planning to conceive during the trial. Participants who did not complete both baseline and end of study Addiction Severity Index Self-Report (ASI) and Timeline Follow-Back (TLFB) questionnaires were also excluded from the present analyses.

### Procedures

The Canadian Research Initiative in Substance Misuse (CRISM) conducted this study at various locations across Canada, namely Calgary (Alberta), Edmonton (Alberta), Montreal (Québec), Sudbury (Ontario), Toronto (Ontario), and Vancouver (British Colombia) (details of sites can be found in Table 1). Recruitment occurred from October 2, 2017 to March 23, 2020. Eligible participants underwent baseline assessments and were randomly assigned in a 1:1 ratio to receive either methadone or BUP/NX, stratified by study sites and lifetime heroin use. OAT initiation adhered to local regulations and Health Canada-approved product monographs. Participants had follow-up visits every two weeks over the course of the study. Due to the COVID-19 pandemic, follow-up assessments were conducted via telephone instead of in person starting March 16, 2020.

**Table 1:** Demographic and baseline characteristics of analyzed participants

Characteristic		MMT (*n* = 64)		BUP/NX (*n* = 50)		TOTAL (*n* = 114)	
Age, mean (SD), years		41.3	10.8	40.1	0.12	40.8	11.1
Female sex, n (%)		19	29.7%	10	20.0%	29	25.4%
Gender, n (%)							
	Man	44	68.8%	39	78.0%	83	72.8%
	Woman	19	29.7%	11	22.0%	30	26.3%
	Transgender	1	1.56%	0	0.0%	1	0.9%
Recruitment site, n (%)							
	CAMH	10	15.6%	13	20.3%	23	20.2%
	CHUM	25	39.1%	20	31.3%	45	39.5%
	CRAN	3	4.7%	2	3.1%	5	4.4%
	ODPC	13	20.3%	4	6.3%	17	14.9%
	PHSC	1	1.6%	0	0.0%	1	0.9%
	RAAC	12	18.8%	11	17.2%	23	20.2%
Prescription drug coverage, n (%)							
	Provincial health insurance	23	35.9%	17	34.0%	40	35.1%
	Pharmacare	6	9.4%	5	10.0%	11	9.7%
	Persons with disabilities	6	9.4%	4	8.0%	10	8.8%
	Private insurance	6	9.4%	4	8.0%	10	8.8%
	Other	13	20.3%	12	24.0%	25	21.9%
	No coverage	10	15.6%	7	14.0%	17	14.9%
	Unknown	0	0.0%	1	2.0%	1	0.9%
Lifetime heroin use, n (%)		41	64.1%	33	66.0%	74	64.9%
Fentanyl use at baseline, n (%)		25	39.1%	15	30.0%	40	35.1%
Highest level of schooling completed, n (%)							
	Incomplete high school	15	23.4%	7	14.0%	22	19.3%
	High school	22	34.4%	19	38.0%	41	36.0%
	Technical/trade school	8	12.5%	6	12.0%	14	12.3%
	Some college/university	8	12.5%	8	16.0%	16	14.0%
	College/university	11	17.2%	10	20.0%	21	18.4%
Ethnicity, n (%)							
	White	46	71.9%	37	74.0%	83	72.8%
	Asian	1	1.6%	0	0.0%	1	0.9%
	Latin American/Hispanic	0	0.0%	1	2.0%	1	0.9%
	Black African	1	1.6%	1	2.0%	1	0.9%
	Black Caribbean	0	0.00%	0	0.0%	1	0.9%
	First Nations	9	14.1%	8	16.0%	17	14.9%
	Metis	1	1.6%	2	4.0%	3	2.6%
	Other	5	7.8%	1	2.0%	6	5.3%
	Choose not to answer	1	1.6%	0	0.0%	1	0.9%
Current living situation, n (%)							
	Very unstable	19	29.7%	9	18.0%	28	24.6%
	A little unstable	11	17.2%	7	14.0%	18	15.8%
	Neither unstable nor stable	9	14.1%	4	8.0%	13	11.4%
	A little stable	5	7.8%	8	16.0%	13	11.4%
	Very stable	18	28.1%	21	42.0%	39	34.2%
	Don’t know	1	1.6%	0	0.0%	1	0.9%
	Choose not to answer	1	1.6%	1	2.0%	2	1.8%
Employment, n (%)		29	45.3%	25	50.0%	54	47.4%
Monthly salary, mean (SD), CAD		617	948	1487	2703	999	1964
Marital status, n (%)							
	Never married	42	65.6%	28	56.0%	70	61.4%
	Married	3	4.7%	1	2.0%	4	3.5%
	In a relationship	7	10.9%	8	16.0%	15	13.2%
	Divorced or separated	10	15.6%	12	24.0%	22	19.3%
	Widowed	2	3.1%	1	2.0%	3	2.6%
Medical comorbidities, n (%)							
	Psychiatric	24	37.5%	19	38.0%	43	37.7%
	Musculoskeletal	18	28.1%	13	26.0%	31	27.2%
	Allergies	17	26.6%	13	26.0%	30	26.3%
	Hepatobiliary	7	10.9%	10	20.0%	17	14.9%
	Respiratory and throat	10	15.6%	11	22.0%	21	18.4%
	Dermatological	7	10.9%	5	10.0%	12	10.5%
	Gastrointestinal	14	21.9%	6	12.0%	20	17.5%
	HCV positive	8	12.5%	6	12.0%	14	12.3%
	Cardiovascular	6	9.4%	2	4.0%	8	7.0%
	Endocrine	7	10.9%	4	8.0%	9	7.9%
	Eye, ear, nose and throat	5	7.8%	1	2.0%	6	5.3%
	Genitourinary	3	4.7%	4	8.0%	7	6.1%
	Neurological (stroke)	1	1.6%	3	6.0%	4	3.5%
	Hematological	1	1.6%	2	4.0%	3	2.6%
	HIV positive	0	0.0%	1	2.0%	1	0.9%
	Immunological	0	0.0%	1	2.0%	1	0.9%
	Neoplasia (tumor)	1	1.6%	0	0.0%	1	0.9%

MMT, Methadone; BUP/NX, buprenorphine/naloxone; HCV, hepatitis C virus; HIV, human immunodeficiency virus; RAAC, Rapid Access Addiction Clinic and Portland Hotel Society Clinic (Vancouver); ODPC, Opioid Dependency Program Clinics (Edmonton and Calgary); CAMH, Centre for Addiction and Mental Health (Toronto); OATC, Ontario Addiction Treatment Centre in Sudbury; CHUM, Centre hospitalier de l’Université de Montréal (Montréal); CRAN, Centre de Recherche et d’Aide pour Narcomane (Montréal)

### Measurements

Participants’ demographic information was collected at baseline and adjusted, if needed, at each follow-up visit (i.e. every two weeks during the study). The study focused on potential life problems, as measured by the ASI questionnaire at baseline and after 24 weeks after treatment initiation. The ASI evaluates issues in medical, psychiatric, employment, alcohol and drug use, legal, and family domains over the past 30 days, scoring from 0 (no issues) to 1 (most severe) [34]. Its reliability and validity are well-established across various clinical settings, with the self-report version proving reliable as well [34–36]. For the purpose of this study, the drug use domain was excluded considering its direct relationship with the independent study variable, opioid use.

The TLFB questionnaire was used to measure OUF. This included all opioid use except OAT. This interview-administered questionnaire was administered at baseline, and subsequently every two weeks during the 24-week study period. For the specific focus of this study, we exclusively considered opioid use reported during the 28 days preceding the baseline assessment, as well as days of use within the last 28 days of the study (including week 22 and week 24 study visits) because these two timepoints aligned with ASI’s measurements availability.

### Statistical analyses

#### Primary analyses

A repeated-measure generalized linear mixed model (GLMM) was used to assess the impact over time in the frequency of days of opioid use on potential life problems. Raw data from baseline and end of study were used as two separate timepoints. An interaction term time*frequency of use (TLFB) was included to consider the change in opioid use over time. The model was adjusted for sex, age, study site, OAT group and lifetime heroin use. Analyses were performed using R version 4.1.2. Two-tailed significance level was set at 0.05.

#### Handling of missing data

Only participants who completed both the ASI and TLFB at both baseline and end of study were included in the present study. In the case of participants who did not complete the TLFB at week 24 but who did complete it at week 22, end of study TLFB for the month was computed by imputing the values of week 23 and 24 with the values of week 21 and 22.

#### Sensitivity analyses

All participants who had baseline data available were included in these analyses, even if their end-of-study data were missing. To address these missing data points, we employed an imputation strategy where we assumed that participants who did not provide end-of-study data had not experienced any changes in their pattern of use or functioning.

#### Post-hoc analyses

- **Effect plot exploratory analyses**.

Effect plot derived from the GLMM obtained from the primary analyses was subsequently analyzed to identify tendencies in OUF and potential life problems in different spheres. For each monthly opioid consumption frequency (ranging from 0 days to 28 days out of a total of 28 days), we plotted the change in potential life problems (ASI score) over time, comparing baseline (week 0) to the end of the study (week 24). This analysis was conducted separately for each ASI domain. This analysis was derived from the GLMM rather than directly from raw participant data. A threshold for the maximum number of days of opioid use associated with a positive trend in potential life problems (indicated by a slope of ASI score above zero) was identified for each ASI domain based on these plots. These thresholds represent the highest number of opioid use days per 28-day period, where the predicted change in the ASI score remains positive, indicating improvement in the specific domain.

b) **Impact of OAT group**.

We conducted post-hoc analyses to assess if the interaction between opioid use and potential life problems differed significantly between OAT groups. A GLMM similar to the one used for primary analyses was used, but the interaction term included was (time*frequency of use)*OAT group to assess for a potential interaction with the treatment group. If the interaction was significant for a given ASI domain, GLMM derived from each OAT subgroup were estimated.

## Results

**Fig. 1:**
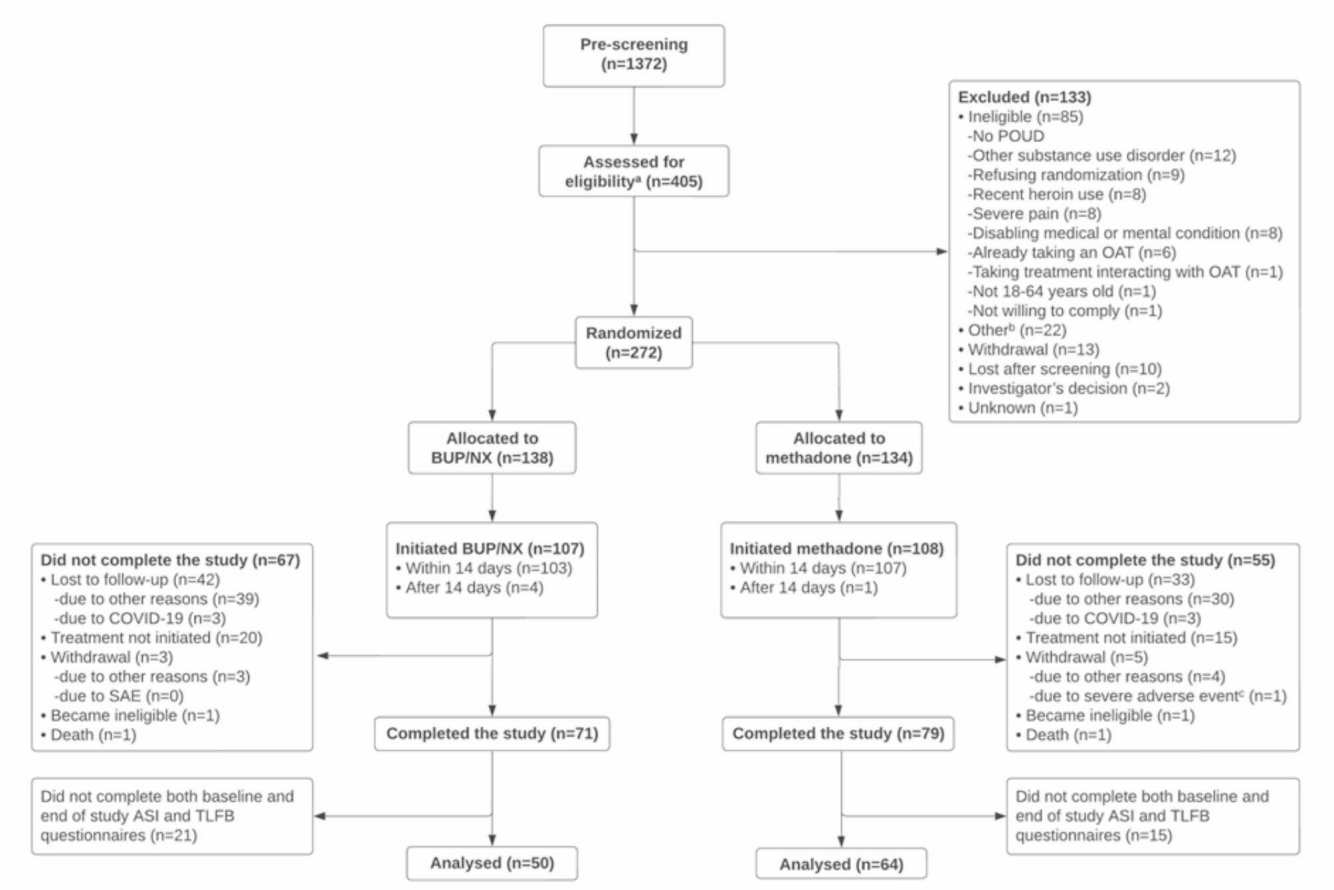
CONSORT flow diagram showing how analyzed participants in the present study were selected and retained. ^a^ Thirteen participants were screened twice but are counted only once in the flowchart. ^b^ Other reasons for exclusions were unspecified (*n* = 14), incomplete screening (*n* = 4), failure to complete baseline visit within a 28-day time window (*n* = 3), and incarceration (*n* = 1). ^c^ One participant attempted suicide and withdrew consent six days later. ASI, Addiction Severity Index Self-Report; TLFB, Timeline Follow-Back; BUP/NX, buprenorphine/naloxone; COVID-19, coronavirus disease 2019; n, number of participants; OAT, opioid agonist treatment; POUD, prescription-type opioid use disorder; SAE, serious adverse events

Figure 1 displays the CONSORT flow diagram, which has been adapted from Jutras-Aswad et al. [20]. A total of 272 eligible participants were randomly assigned to receive either methadone (*n* = 134, 49.3%) or BUP/NX (*n* = 138, 50.7%). Out of these participants, 158 individuals (70 in the methadone group; 88 in the BUP/NX group) did not provide both baseline and end of study ASI and TLFB questionnaires and were consequently excluded from the analyses, leaving 114 participants in the analyzed sample. A comparison of baseline characteristics of included and excluded participants can be found in Supplemental Table 1. In summary, compared to participants excluded from the study, those included exhibited a statistically significant higher tendency being of male sex, having been recruited at the CHUM/CAMH sites, being of white ethnicity, not using fentanyl, having more stable housing, being employed (and having higher monthly salary), and having gastrointestinal comorbidities.

Participants’ demographic and baseline characteristics can be found in Table 1. In short, most participants were middle-aged adults (mean ± standard deviation = 40.8 ± 11.1 years), male (74.6%), white (72.8%), and were never married (61.4%). More than one-third reported having very stable housing (34.2%), but a quarter reported very unstable housing (24.6%). About half were employed (47.4%) and the mean monthly salary was 999 ± 1964 CAD. The most common education level reported was high school degree (36.0%), but about a fifth of participants had not completed high school (19.3%) and a fifth had a college diploma (18.4%). A large proportion of participants had psychiatric (37.7%) or musculoskeletal (27.2%) comorbidities. Two-thirds of participants had previously used heroin at least once (64.9%) and a third used fentanyl at baseline (35.1%).

**Fig. 2:**
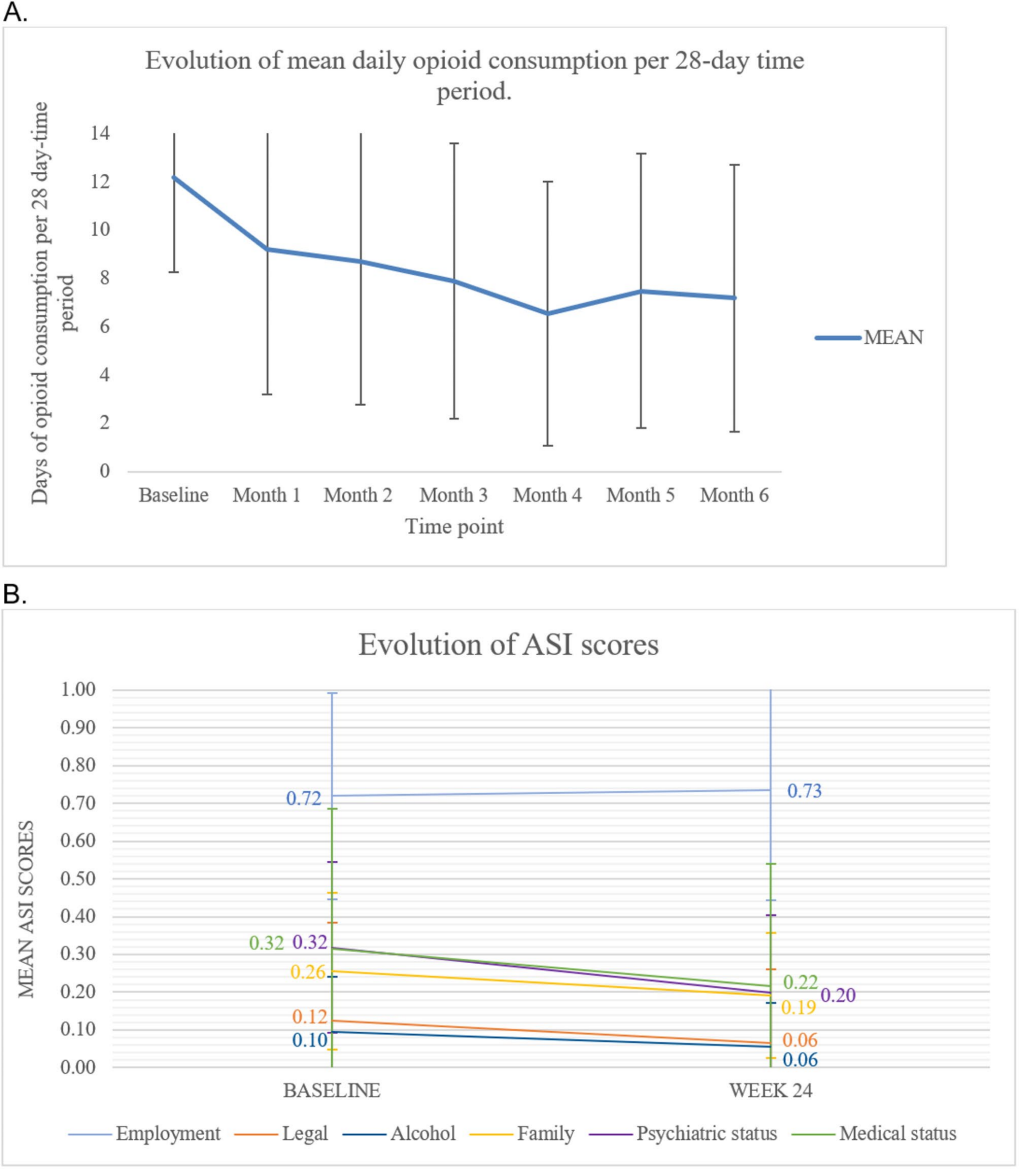
Evolution of past 28-day opioid consumption and ASI scores after opioid agonist treatment initiation. **A**. Panel A shows participants’ mean past 28-day opioid use throughout the course of the study. Opioid use was self-reported every two weeks using the Timeline Follow-Back (TLFB) questionnaire. Error bars indicate standard deviation. **B**. Panel B shows participants’ mean addiction severity index (ASI) scores at baseline and at the end of the study (week 24) for the following spheres: employment, family relationships, legal, psychiatric status, alcohol use, medial status. Error bars indicate standard deviation

Figure 2 shows that participants’ daily opioid consumption was decreased at the end of the study after the introduction of OAT. At baseline, the participants’ mean opioid use over the past 28 days (±standard deviation) was 12.2±3.9 days, which decreased to 7.2±5.5 days at the end of the study. Fifty-six participants were abstinent in the last 4 weeks of the study. Similarly, Fig. 2 also show that mean ASI scores decreased (meaning that participants’ potential life problems decreased) from baseline to the end of the study for medical status, psychiatric status, family situation and relationships, legal status and alcohol problem severity, but not for employment. As seen in Table 2, this change in ASI over time was significant (*p* < 0.05) for all ASI domains, except for employment.

**Table 2:** Association between potential life problems and frequency of daily opioid consumption over time: results from the generalized linear mixed model

	Estimate (beta)	Confidence interval	*p*-value
**Employment**			
time	-0.0559	(-0.1705, 0.0587)	0.337
consumption frequency	-0.0122	(-0.0300, 0.0055)	0.176
time*consumption frequency	0.0051	(-0.0039, 0.0142)	0.264
**Medical status**			
time	-0.2776	(-0.4889, -0.0663)	**0.010**
consumption frequency	-0.0277	(-0.0597, 0.0043)	0.089
time*consumption frequency	0.0167	(0.0001, 0.0334)	**0.049**
**Psychiatric status**			
time	-0.2430	(-0.3648, -0.1212)	**< 0.001**
consumption frequency	-0.0193	(-0.0379, -0.0007)	**0.042**
time*consumption frequency	0.0115	(0.0019, 0.0211)	**0.019**
**Family status**			
time	-0.1269	(-0.2447, -0.0092)	**0.035**
consumption frequency	-0.0107	(-0.0286, 0.0071)	0.238
time*consumption frequency	0.0044	(-0.0049, 0.0138)	0.352
**Legal status**			
time	-0.1925	(-0.3352, -0.0497)	**0.008**
consumption frequency	-0.0216	(-0.0432, 0.000)	0.050
time*consumption frequency	0.0112	(-0.0000, 0.0225)	0.050
**Alcohol problems**			
time	-0.1622	(-0.2398, -0.0845)	**< 0.001**
consumption frequency	-0.0197	(-0.0316, -0.0079)	**0.001**
time*consumption frequency	0.0102	(0.0041, 0.0163)	**0.001**

The main results of our GLMM assessing the interaction between OUF and time on potential life problems can be found in Table 2. A decrease in OUF over time was significantly associated with fewer problems related to medical status (*p* = 0.049), psychiatric status (*p* = 0.019), and alcohol problem severity (*p* = 0.001). The interaction was non-significant for employment (*p* = 0.264), family status (*p* = 0.352) and legal status (*p* = 0.050). Sensitivity analyses, which included all participants with baseline data (*n* = 259) and imputed missing end-of-study data, yielded consistent results except for the relationship between opioid consumption frequency at baseline and psychiatric status that was not significant. These results can be found in Supplemental Table 2.

Post-hoc analyses of the impact of OAT group on our interaction of interest can be found in Supplemental Table 3. The interaction between OUF and time did not differ significantly between treatment groups for most ASI domains (*p* < 0.05) except family status (*p* = 0.030), where the interaction was significant for the group treated with methadone (*p* = 0.029) but not for the group treated with BUP/NX (*p* = 0.258).

**Fig. 3:**
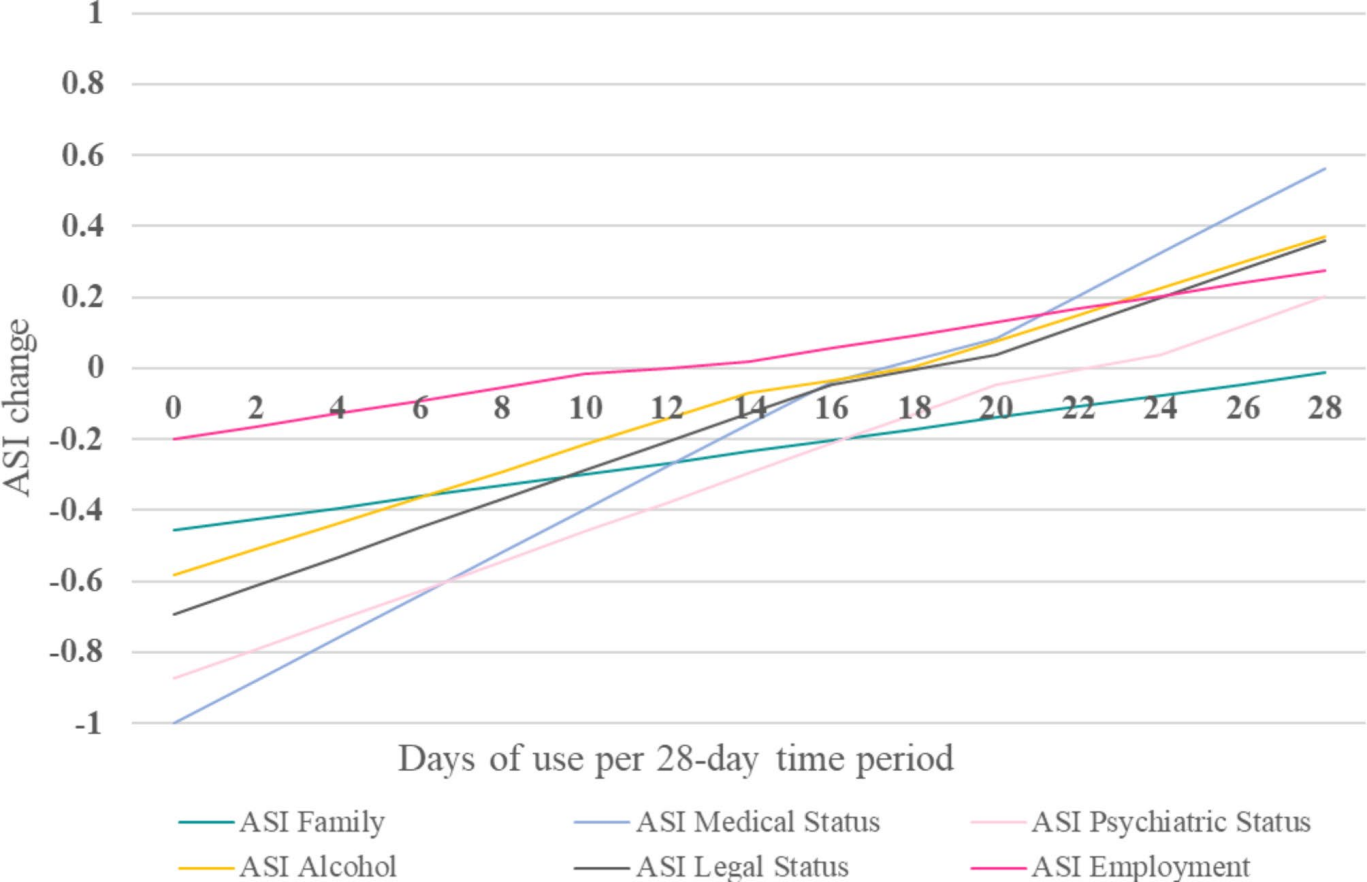
Potential life problems change over time for given opioid consumption frequency levels per 28-day period. Effect plot was derived from the generalized linear mixed model. Opioid use was self-reported every two weeks using the Timeline Follow-Back (TLFB) questionnaire. ASI change represents the change in ASI score for each domain from baseline (week 0) to the end of the study (week 24). ASI = Addiction severity index

**Fig. 4:**
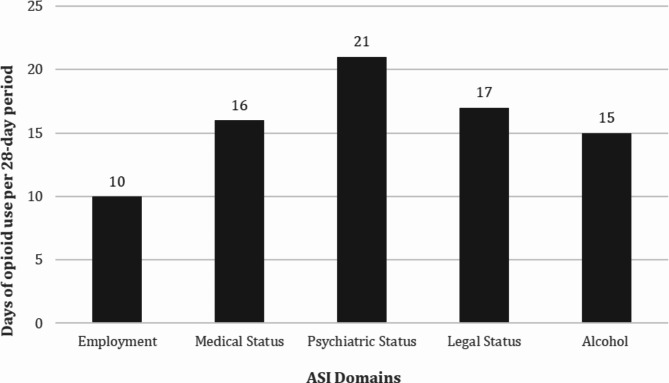
Opioid use frequency thresholds required for improvement in potential life problems. Maximum number of days of opioid use per 28-day period associated with a positive trend in potential life problems was identified for each ASI domain based on effect plots (Fig. 3, S1) derived from the generalized linear mixed model. A positive trend in potential life problems was defined by a slope of ASI score above zero in the effect plots. The family status ASI domain has not been included because it tended to improve no matter the frequency of opioid use. ASI = Addiction severity index

Figure 3 presents the post hoc analysis illustrating the changes in potential life problems over time across different levels of opioid consumption frequency. For all ASI domains, ASI score tended to improve for lower opioid use frequency per four-week time period and tended to deteriorate for higher opioid use frequency, except for ASI family that improved for all frequencies of use. Detailed effect plots illustrating the change for each level of consumption can be found in Supplemental Fig. 1. As seen in Fig. 4, the maximal number of days of opioid use per 28 day-period for which an improvement in potential life problems over the study period could be seen –albeit not statistically significant for all domains as discussed in the previous paragraph– was 21 for psychiatric status, 17 for legal status, 16 for medical status, 15 for alcohol problem severity and 10 for employment.

## Discussion

To our knowledge, this is the first study to examine the association between OUF during OAT and potential life problems in individuals with POUD. Initiation of OAT was associated with improvement in all life areas except for employment. A reduction in opioid use was associated with an improvement in medical status, psychiatric status and alcohol problem severity over the course of study participation, but not in employment, family relationships and legal status. Although 56 participants achieved abstinence in the last month of the study, qualitative observations of data revealed improvements in potential life problems not only among individuals who achieved complete abstinence but also among those who reduced opioid use without abstaining completely.

As previously highlighted, the only domain that did not show statistically significant improvement during OAT was employment. A possible explanation is that it may take more than 6 months (duration of the present study) for individuals who have an OUD to return to the workforce. Indeed, it has been shown that unemployment often persists during treatment and remission for individuals with OUD [37–39]. Barriers to employment after treatment for substance use disorder include lack of employment skills, having a criminal record, big gaps in employment history, employers’ stigma toward substance use disorder history and need for daily witnessed ingestion in the case of methadone [40–42]. Longer term assessment may be necessary to detect a potential positive impact of OAT on this domain, if any.

The association between decrease in opioid use and the improved medical status, psychiatric status, and alcohol-related outcomes may be explained by several factors. Lowering opioid use reduces high-risk behaviors (for example intravenous drug use), potentially lessening associated medical complications such as HIV, hepatitis, cellulitis, thrombosis, and endocarditis [41, 43]. In addition, improvement in medical symptoms could stem from the decrease in opioid-induced hyperalgesia seen in regular opioid use [44] and from a reduction in occurrence and severity of cycles of intoxication interspersed with withdrawal periods (marked by various uncomfortable symptoms, including muscle aches, fever, nausea, vomiting and diarrhea) [45, 46]. Regarding the improvement of psychiatric condition, prescription opioid use is highly comorbid with psychiatric disorders, especially major depressive disorder and anxiety disorders [47–50]. The relationship between opioid use and mental health is intricate, likely involving a bidirectional interaction where each factor may influence and exacerbate the other [51, 52]. Opioid receptors, particularly the kappa opioid receptor, are recognized for their involvement in the pathophysiology of mood disorders [47–50]. Opioids modify brain neurotransmitter circuits, including serotonin and dopamine, crucial for various psychiatric disorders [53]. Moreover, reducing the time devoted to opioid use has the potential to free up resources and energy, enabling individuals to invest in other aspects of life. Our findings align with the demonstration of Bastien et al. that improvements in depressive symptoms during OAT were partially mediated by a reduction in opioid use [54].

The association between reduced opioid use and decreased alcohol use found in this study is consistent with the literature that shows a clear interaction between OUD and alcohol use disorder, and underlying neuromolecular pathways [55]. Cross-tolerance has been observed in patients using both substances [56, 57]. Therefore, it is plausible that decreased opioid use would reduce positive reinforcement signaling pathways involved in both opioid and alcohol use disorder. Moreover, reduced frequency of recurrent intoxication and withdrawals, which are often linked to symptoms like anxiety, irritability, and overall emotional dysregulation, may diminish individuals’ need to seek relief through self-medication with substances like alcohol. Interestingly, the baseline data show that reduced opioid use is associated with more psychiatric and alcohol use problems. This may reflect that those who experience more severe psychiatric symptoms or conditions may not be able to sustain high frequency opioid use, while others may also have more complex pattern of co-use with alcohol, not solely focused on opioid consumption. Notwithstanding these counterintuitive cross-sectional findings at baseline, the positive significant interaction term reveals the benefit of changing opioid frequency over time, even in the context of diverse profile of co-occurring use of opioid and other substances, as well as comorbid conditions.

In contrast, the absence of significant association for a decrease in opioid use and employment, family situation and legal status may be explained by the involvement of external factors in those spheres. Family relationships and employment involve not only the participant, but also other figures (e.g.: family members, employers). It may take longer than the study’s six-month duration for relationships to improve and rebuild, especially if the participant had been using opioids for a long time. As discussed previously, unemployment often persists during and after treatment of OUD. Similarly, legal causes for past charges may still be active and may take months or even years to resolve. Contrastingly to external factors, shared vulnerabilities, such as fragile self-esteem, trauma, or a history of adverse childhood experiences, can impact not only the ability to maintain healthy relationships but also contribute to challenges in securing stable employment and navigating legal matters.

For all domains except family situation and relationships, lower OUF was associated with reduced potential life problems from the beginning to the end of the study. Unsurprisingly, the consumption frequency threshold to see improvement in the employment domain is lower than what is seen with the other domains; employment requires not only minimum health requirements with regards to psychiatric and physical conditions but also other attributes such as preserved cognitive functions, which can be altered by drug use. Family situation and relationships tended to improve no matter the OUF. A potential explanation for the divergent trend observed in the family situation and relationships domain, as opposed to other domains, is that families may shift their attitudes when an individual chooses to engage in treatment for their OUD, regardless of the outcome of that treatment. In short, we can conclude that 10 days or less of opioid use per 28 day-period (or on average 2.5 days per week) was required in our study to see an improving trend in potential life problems in all life spheres, but improvement in some spheres was seen starting at 21 days of opioid use per 28 day-period (or on average 5 days per week).

In a harm reduction perspective, reduced patterns of use other than abstinence have been associated with improved functioning in people with other types of substance use disorder. For example, in individuals undergoing treatment for cocaine use disorder, low frequency of cocaine use (estimated to be at 1 day/week) during treatment was associated with better functioning at 6 months than was persistent frequent use (estimated to be at 4 days/week) in the psychological, family, employment, and legal domains [58]. Furthermore, functioning at 12 months after treatment did not differ between low frequency of cocaine use and abstinence during treatment [58]. For alcohol-use disorder, low-risk drinking (as defined by the FDA [59] as no heavy drinking days, i.e. no more than 3 standard drinks per day for women and no more than 4 for men) has been associated with similar psychosocial function and health outcomes compared to abstinence while in treatment [60–63]. Our study’s alignment with findings in cocaine and alcohol use disorders suggests a shared impact of reduced substance use on psychosocial functioning across various substances. This broader pattern emphasizes the importance of flexibility in treatment approaches, recognizing that different individuals may benefit from tailored strategies rather than strictly adhering to abstinence-based models.

Among its strengths, this study stands out as one of the few initiatives to assess the impact of opioid use frequency on associated substance use problems, while its pragmatic design facilitates its translation in clinical settings. However, several limitations should be noted. First, of the initial 259 participants who completed the baseline ASI questionnaire, only 114 completed the end-of-study questionnaire and were thus included in our analyses. The nature of our outcomes (i.e., likelihood of missing not at random) also limited the imputation strategies that could be used for analysis. This and the high rate of missing data necessitate caution when interpreting our findings. Nevertheless, the sensitivity analyses, which addressed the missing data by imputing end-of-study values, yielded consistent results. This consistency suggests that the observed associations remain robust despite the data gaps. It is important to note, however, that participants who remained in the study had better baseline functioning compared to those who were excluded, potentially leading to an overrepresentation of this subgroup in our analyses. Moreover, the assessment of potential life problems relied on a self-reported measure of problem severity, lacking objective indicators. The ASI instrument, used to assess potential life problems in this study, has been noted in previous research [64, 65] to have limited sensitivity in detecting changes over time. However, the fact that significant reductions in life problems were observed despite this limitation suggests that the positive outcomes related to decreased opioid use frequency may be particularly robust and meaningful. Furthermore, we solely assessed OUF, lacking data on daily consumption quantities, potentially overlooking cases where patients reduced use frequency but increased daily intake. In addition, days of use were also self-reported, since urine tests were only done every two weeks and have a detection window for opioids of only 1 to 4 days [66]. Another limitation is our focus on the actual number of days of use per month as the therapeutic outcome. While this provided valuable insights for treatment goals, future research could explore how specific reductions in the number of days of use contribute to improvements in potential life problems. Additionally, the identified consumption thresholds did not always result in statistically significant changes across domains, highlighting the need for further research to determine the number of opioid use days associated with meaningful improvements in potential life problems. Future research should explore prospective associations between reductions in opioid use and improvements in long-term functioning, building on concurrent associations observed in this study. Finally, when extrapolating the findings of this trial to a broader population, it is important to consider the exclusion of potential participants with chronic pain conditions necessitating opioid-based pain management. In addition, our study exclusively involved Canadian sites and the generalization of our findings to other countries may be limited.

## Conclusions

In conclusion, this study suggests that a reduction in the frequency of opioid consumption during OAT is associated with a reduction in potential life problems. Notably, our findings suggest that a threshold of 21 days of opioid use per 28 day-period (or 5 days / week) was necessary to observe improvements in some spheres and 10 days or less of opioid use per 28 day-period (or 2.5 days / week) was required to see an improving trend in potential life problems in all life domains assessed. This study aligns with a growing body of evidence suggesting that a harm reduction perspective, allowing for patterns of substance use other than abstinence, can lead to improved functioning and well-being. It underscores the importance of flexibility in treatment models, recognizing that diverse individuals may benefit from tailored strategies that go beyond rigid abstinence-based expectations. As our understanding of addiction continues to evolve, such insights are crucial for informing evidence-based interventions and ultimately better addressing the complexities of the opioid epidemic.

Tables and figures have been uploaded in separate files.

## Electronic supplementary material

Below is the link to the electronic supplementary material.

Supplementary Material 1

Supplementary Material 2

Supplementary Material 3

Supplementary Material 4

## Data Availability

The datasets analysed during the current study are available from the corresponding author on reasonable request.

## Abbreviations

OUD: Opioid use disorder
POUD: Prescription-type opioid use disorder
OAT: Opioid Agonist Treatment
BUP/NX: Buprenorphine/naloxone
ASI: Addiction Severity Index Self-Report
TLFB: Timeline Follow-Back
