# Supplementary material for: Opioid consumption frequency and its associations with potential life problems during opioid agonist treatment in individuals with prescription-type opioid use disorder: exploratory results from the OPTIMA Study
Source: Harm Reduct J. 2025 Feb 8;22:14. doi: 10.1186/s12954-025-01157-4 (PMC11806552; doi:10.1186/s12954-025-01157-4)
Supplement: Supplementary file 1 — Supplementary Material 1 [file 12954_2025_1157_MOESM1_ESM.docx]

**Supplemental Table 1.** Demographic and baseline characteristics of included compared to excluded participants

| Characteristic | | Included  (n=114) | | Excluded  (n=158) | | p-value^a^ |
| --- | --- | --- | --- | --- | --- | --- |
| Age, mean (SD), years | | 40.8 | 11.1 | 37.6 | 10 | 0.014 |
|  |  |  |  |  |  |  |
| Female sex, n (%) | | 29 | 25.4% | 64 | 44.3% | 0.014 |
| Gender, n (%) |  |  |  |  |  | 0.052 |
|  | Man | 83 | 72.8% | 93 | 58.9% |  |
|  | Woman | 30 | 26.3% | 64 | 40.5% |  |
|  | Transgender | 1 | 0.9% | 1 | 0.6% |  |
| Recruitment site, n (%) | |  |  |  |  | <0.001 |
|  | CAMH | 23 | 20.2% | 26 | 16.5% |  |
|  | CHUM | 45 | 39.5% | 20 | 12.7% |  |
|  | CRAN | 5 | 4.4% | 2 | 1.3% |  |
|  | ODPC | 17 | 14.9% | 65 | 41.1% |  |
|  | PHSC | 1 | 0.9% | 8 | 5.1% |  |
|  | RAAC | 23 | 20.2% | 37 | 23.4% |  |
| Prescription drug coverage, n (%) | |  |  |  |  | 0.173 |
|  | Provincial health insurance | 40 | 35.1% | 36 | 22.8% |  |
|  | Pharmacare | 11 | 9.7% | 24 | 15.2% |  |
|  | Persons with disabilities | 10 | 8.8% | 14 | 8.9% |  |
|  | Private insurance | 10 | 8.8% | 4 | 2.5% |  |
|  | Other | 25 | 21.9% | 43 | 27.2% |  |
|  | No coverage | 17 | 14.9% | 31 | 19.6% |  |
|  | Unknown | 1 | 0.9% | 6 | 3.8% |  |
| Lifetime heroin use, n (%) | | 74 | 64.9% | 113 | 71.5% | 0.304 |
| Fentanyl use at baseline, n (%) | | 40 | 35.1% | 110 | 69.6% | <0.001 |
| Highest level of schooling completed, n (%) | |  |  |  |  | 0.125 |
|  | Incomplete high school | 22 | 19.3% | 28 | 17.7% |  |
|  | High school | 41 | 36.0% | 77 | 48.7% |  |
|  | Technical/trade school | 14 | 12.3% | 11 | 7.0% |  |
|  | Some college/university | 16 | 14.0% | 16 | 10.1% |  |
|  | College/university | 21 | 18.4% | 25 | 15.8% |  |
|  | Choose not to answer | 0 | 0.0% | 1 | 0.6% |  |
| Ethnicity, n (%) |  |  |  |  |  | <0.001 |
|  | White | 83 | 72.8% | 100 | 63.3% |  |
|  | Asian | 1 | 0.9% | 3 | 1.9% |  |
|  | Latin American/Hispanic | 1 | 0.9% | 0 | 0.0% |  |
|  | Middle Eastern | 0 | 0.0% | 2 | 1.3% |  |
|  | Black African | 1 | 0.9% | 1 | 0.6% |  |
|  | Black Caribbean | 1 | 0.9% | 1 | 0.6% |  |
|  | First Nations | 17 | 14.9% | 29 | 18.4% |  |
|  | Metis | 3 | 2.6% | 10 | 6.3% |  |
|  | Other | 6 | 5.3% | 10 | 6.3% |  |
|  | Choose not to answer | 1 | 0.9% | 2 | 1.3% |  |
| Current living situation, n (%) | |  |  |  |  | 0.019 |
|  | Very unstable | 28 | 24.6% | 57 | 36.1% |  |
|  | A little unstable | 18 | 15.8% | 22 | 13.9% |  |
|  | Neither unstable nor stable | 13 | 11.4% | 9 | 5.7% |  |
|  | A little stable | 13 | 11.4% | 29 | 18.4% |  |
|  | Very stable | 39 | 34.2% | 36 | 22.8% |  |
|  | Don't know | 1 | 0.9% | 5 | 3.2% |  |
|  | Choose not to answer | 2 | 1.8% | 0 | 0.0% |  |
| Employment, n (%) | | 54 | 47.4% | 46 | 29.0% | 0.003 |
| Monthly salary, mean (SD), CAD | | 999 | 1964 | 347 | 887 | <0.001 |
| Marital status, n (%) | |  |  |  |  | 0.993 |
|  | Never married | 70 | 61.4% | 102 | 64.6% |  |
|  | Married | 4 | 3.5% | 6 | 3.8% |  |
|  | In a relationship | 15 | 13.2% | 19 | 12.0% |  |
|  | Divorced or separated | 22 | 19.3% | 28 | 17.7% |  |
|  | Widowed | 3 | 2.6% | 3 | 1.9% |  |
| Medical comorbidities, n (%) | |  |  |  |  |  |
|  | Psychiatric | 43 | 37.7% | 79 | 50.0% | 0.059 |
|  | Musculoskeletal | 31 | 27.2% | 35 | 22.2% | 0.416 |
|  | Allergies | 30 | 26.3% | 34 | 21.7% | 0.431 |
|  | Hepatobiliary | 17 | 14.9% | 34 | 21.7% | 0.213 |
|  | Respiratory and throat | 21 | 18.4% | 29 | 18.4% | 1.000 |
|  | Dermatological | 12 | 10.5% | 22 | 14.1% | 0.491 |
|  | Gastrointestinal | 20 | 17.5% | 12 | 7.6% | 0.019 |
|  | HCV positive | 14 | 12.3% | 18 | 11.4% | 0.157 |
|  | Cardiovascular | 8 | 7.0% | 11 | 7.0% | 1.000 |
|  | Endocrine | 9 | 7.9% | 7 | 4.4% | 0.349 |
|  | Eye, ear, nose and throat | 6 | 5.3% | 9 | 5.7% | 1.000 |
|  | Genitourinary | 7 | 6.1% | 7 | 4.4% | 0.725 |
|  | Neurological (stroke) | 4 | 3.5% | 6 | 3.8% | 1.000 |
|  | Hematological | 3 | 2.6% | 6 | 3.8% | 0.837 |
|  | HIV positive | 1 | 0.9% | 0 | 0.0% | 0.663 |
|  | Immunological | 1 | 0.9% | 2 | 1.3% | 1.000 |
|  | Neoplasia (tumor) | 1 | 0.9% | 2 | 1..3% | 1.000 |

MMT, Methadone; BUP/NX, buprenorphine/naloxone; HCV, hepatitis C virus; HIV, human immunodeficiency virus; RAAC, Rapid Access Addiction Clinic and Portland Hotel Society Clinic (Vancouver); ODPC, Opioid Dependency Program Clinics (Edmonton and Calgary); CAMH, Centre for Addiction and Mental Health (Toronto); OATC, Ontario Addiction Treatment Centre in Sudbury; CHUM, Centre hospitalier de l’Université de Montréal (Montréal); CRAN, Centre de Recherche et d’Aide pour Narcomane (Montréal).

^a^ P-values were calculated using chi-square tests for categorical variables and analysis of variance (ANOVA) for continuous variables.
