# Supplementary material for: Opioid consumption frequency and its associations with potential life problems during opioid agonist treatment in individuals with prescription-type opioid use disorder: exploratory results from the OPTIMA Study
Source: Harm Reduct J. 2025 Feb 8;22:14. doi: 10.1186/s12954-025-01157-4 (PMC11806552; doi:10.1186/s12954-025-01157-4)
Supplement: Supplementary file 2 — Supplementary Material 2 [file 12954_2025_1157_MOESM2_ESM.docx]

**Supplemental Table 2.** Association between potential life problems and frequency of daily opioid consumption over time: sensitivity analysis using generalized linear mixed models with imputed data.

|  | **Estimate (beta)** | **Confidence interval** | **p-value** |
| --- | --- | --- | --- |
| **Employment** |  |  |  |
| time | -0.0220 | -0.0670 – 0.0231 | 0.339 |
| consumption frequency | -0.0064 | -0.0135 – 0.0007 | 0.076 |
| time*consumption frequency | 0.0020 | -0.0015 – 0.0056 | 0.266 |
| **Medical status** |  |  |  |
| time | -0.1292 | -0.2223 – -0.0361 | **0.007** |
| consumption frequency | -0.0109 | -0.0253 – 0.0036 | 0.140 |
| time*consumption frequency | 0.0080 | 0.0007 – 0.0154 | **0.033** |
| **Psychiatric status** |  |  |  |
| time | -0.1092 | -0.1631 – -0.0553 | **<0.001** |
| consumption frequency | -0.0067 | -0.0151 – 0.0017 | 0.116 |
| time*consumption frequency | 0.0054 | 0.0012 – 0.0097 | **0.013** |
| **Family status** |  |  |  |
| time | -0.0627 | -0.1144 – -0.0110 | **0.017** |
| consumption frequency | -0.0073 | -0.0153 – 0.0007 | 0.073 |
| time*consumption frequency | 0.0026 | -0.0015 – 0.0066 | 0.220 |
| **Legal status** |  |  |  |
| time | -0.0690 | -0.1316 – -0.0064 | **0.031** |
| consumption frequency | -0.0057 | -0.0155 – 0.0040 | 0.249 |
| time*consumption frequency | 0.0040 | -0.0010 – 0.0089 | 0.116 |
| **Alcohol problems** |  |  |  |
| time | -0.0680 | -0.1017 – -0.0343 | **<0.001** |
| consumption frequency | -0.0082 | -0.0134 – -0.0030 | **0.002** |
| time*consumption frequency | 0.0042 | 0.0016 – 0.0069 | **0.002** |
|  |  |  |  |
