# Supplementary material for: Opioid consumption frequency and its associations with potential life problems during opioid agonist treatment in individuals with prescription-type opioid use disorder: exploratory results from the OPTIMA Study
Source: Harm Reduct J. 2025 Feb 8;22:14. doi: 10.1186/s12954-025-01157-4 (PMC11806552; doi:10.1186/s12954-025-01157-4)
Supplement: Supplementary file 3 — Supplementary Material 3 [file 12954_2025_1157_MOESM3_ESM.docx]

**Supplemental Table 3.** Impact of opioid agonist treatment group on the association between potential life problems and frequency of daily opioid consumption over time: results from the generalized linear mixed model.

MMT, Methadone; BUP/NX, buprenorphine/naloxone; oat, opioid agonist treatment.

|  |  |  |  |
| --- | --- | --- | --- |
| **Employment** | **Estimate (beta)** | **Confidence interval** | **p-value** |
| (time*consumption frequency) * oat | 0.0132 | (-0.0054, 0.0318) | 0.163 |
| **Medical status** |  |  |  |
| (time*consumption frequency) * oat | 0.0197 | (-0.0140, 0.0534) | 0.250 |
| **Psychiatric status** |  |  |  |
| (time*consumption frequency) * oat | 0.0178 | (-0.0018, 0.0374) | 0.074 |
| **Family status** |  |  |  |
| (time*consumption frequency) * oat | 0.0208 | (0.0020, 0.0396) | **0.030** |
| **Family status** – Subgroup analyses  MMT – (time*consumption frequency)  BUP/NX – (time*consumption frequency)  **Legal status** | 0.0129  -0.0090 | (0.0013, 0.0245)  (-0.0248, 0.0067) | **0.029**  0.258 |
| (time*consumption frequency) * oat | 0.0038 | (-0.0191, 0.0267) | 0.743 |
| **Alcohol problems** |  |  |  |
| (time*consumption frequency) * oat | -0.0082 | (-0.0206, 0.0042) | 0.193 |
|  |  |  |  |
