# Supplementary material for: Opioid consumption frequency and its associations with potential life problems during opioid agonist treatment in individuals with prescription-type opioid use disorder: exploratory results from the OPTIMA Study
Source: Harm Reduct J. 2025 Feb 8;22:14. doi: 10.1186/s12954-025-01157-4 (PMC11806552; doi:10.1186/s12954-025-01157-4)
Supplement: Supplementary file 4 — Supplementary Material 4 [file 12954_2025_1157_MOESM4_ESM.docx]

| 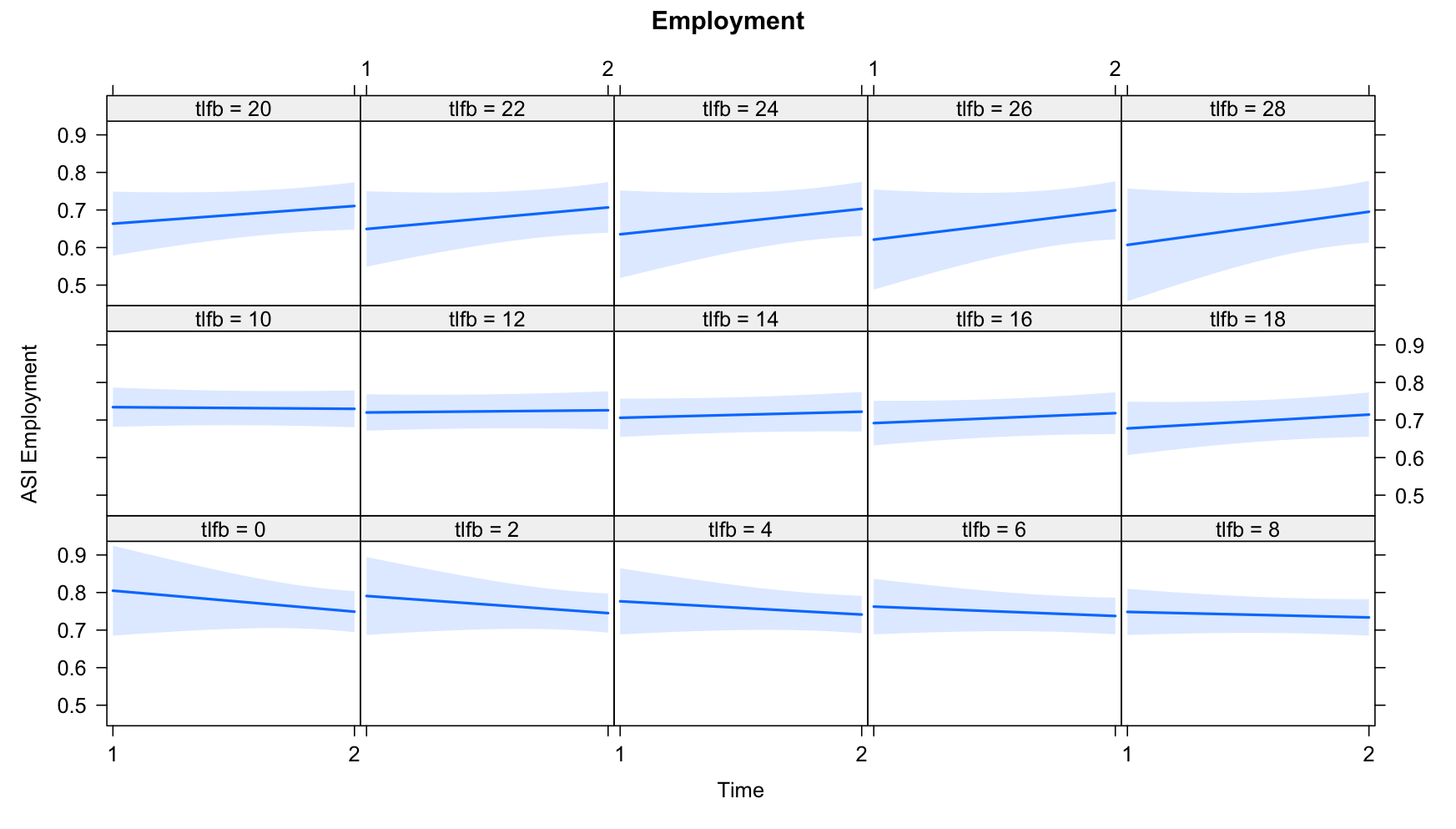 | **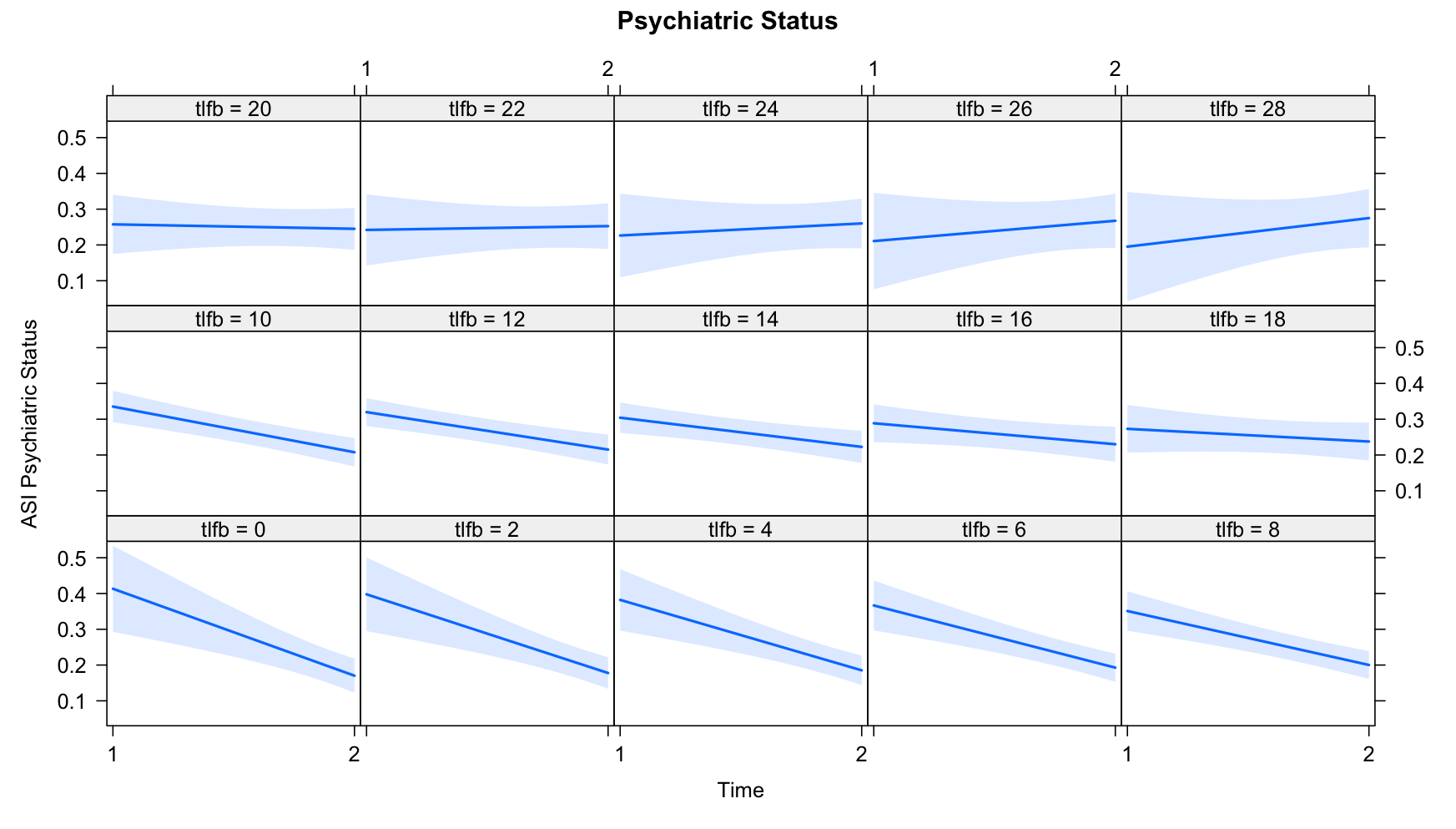** |
| --- | --- |
| **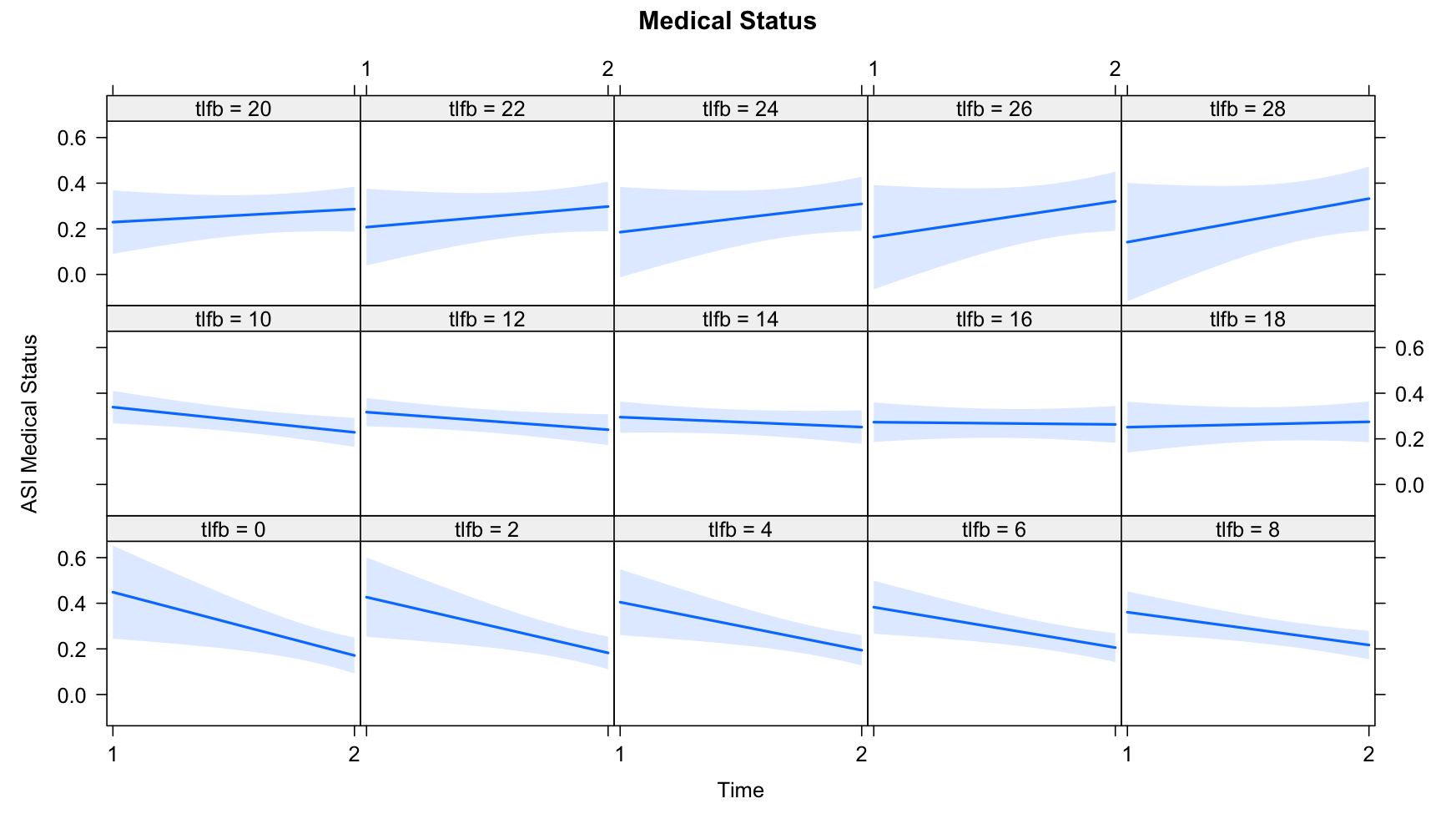** | **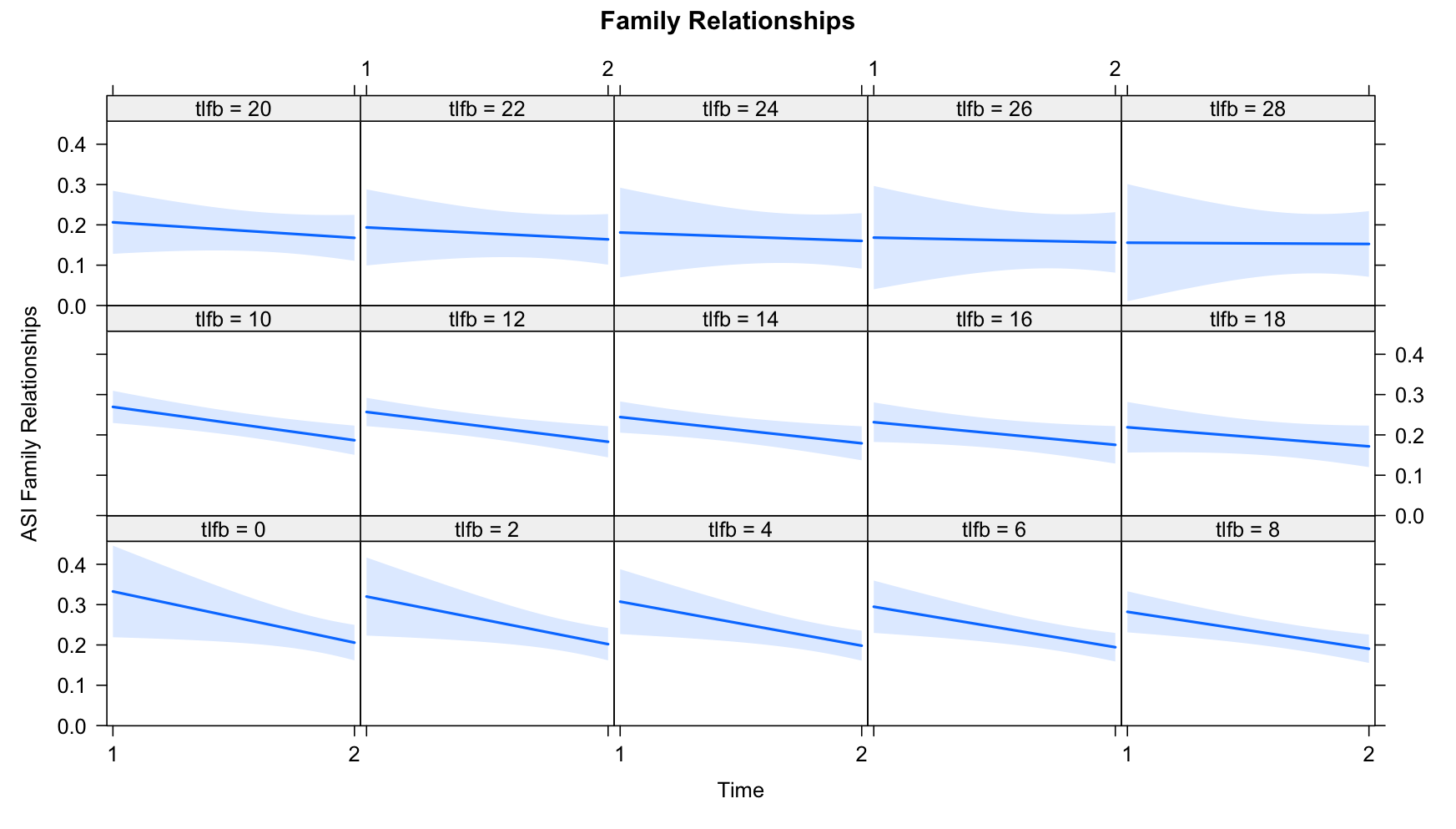** |
| **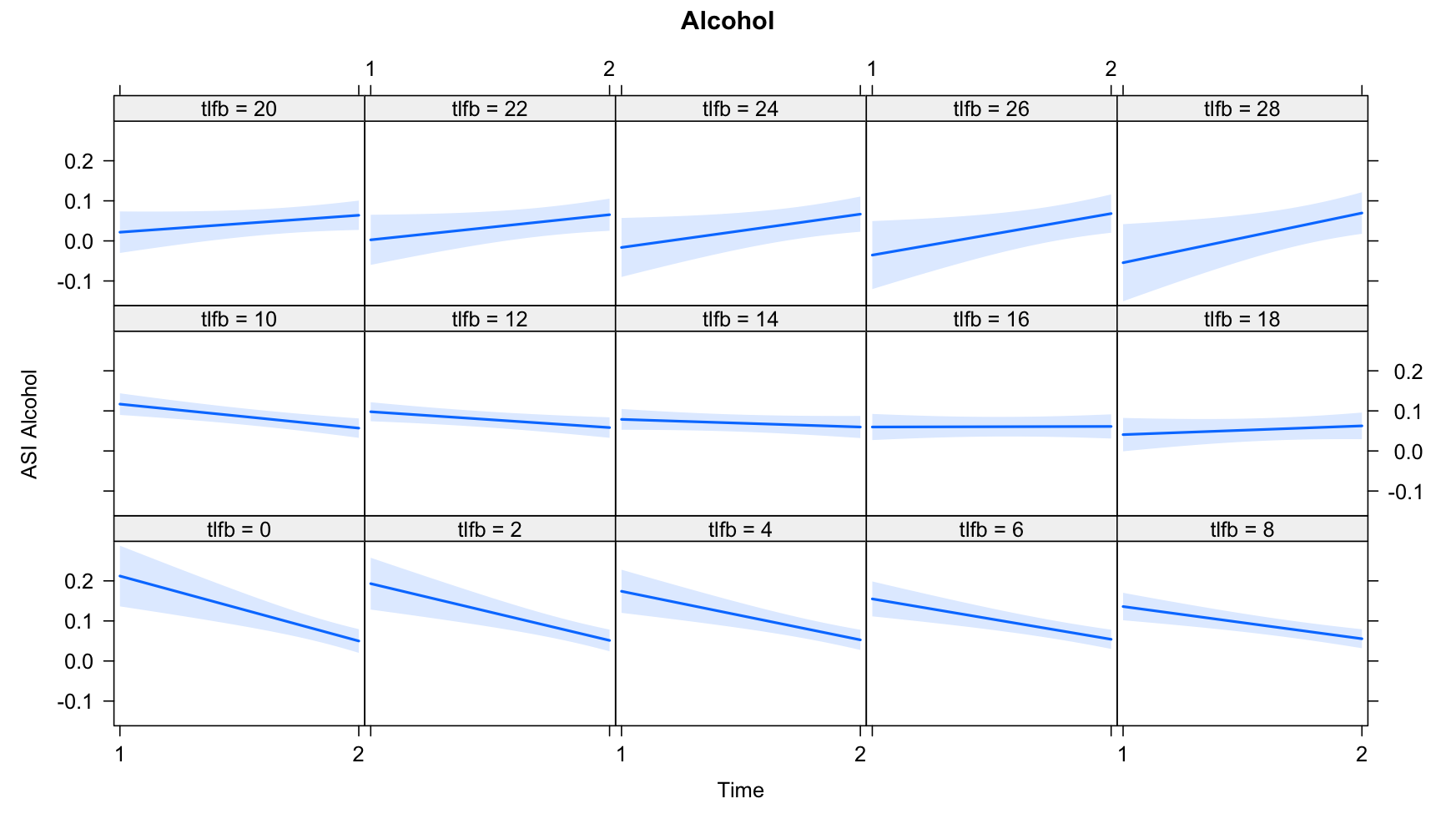** | **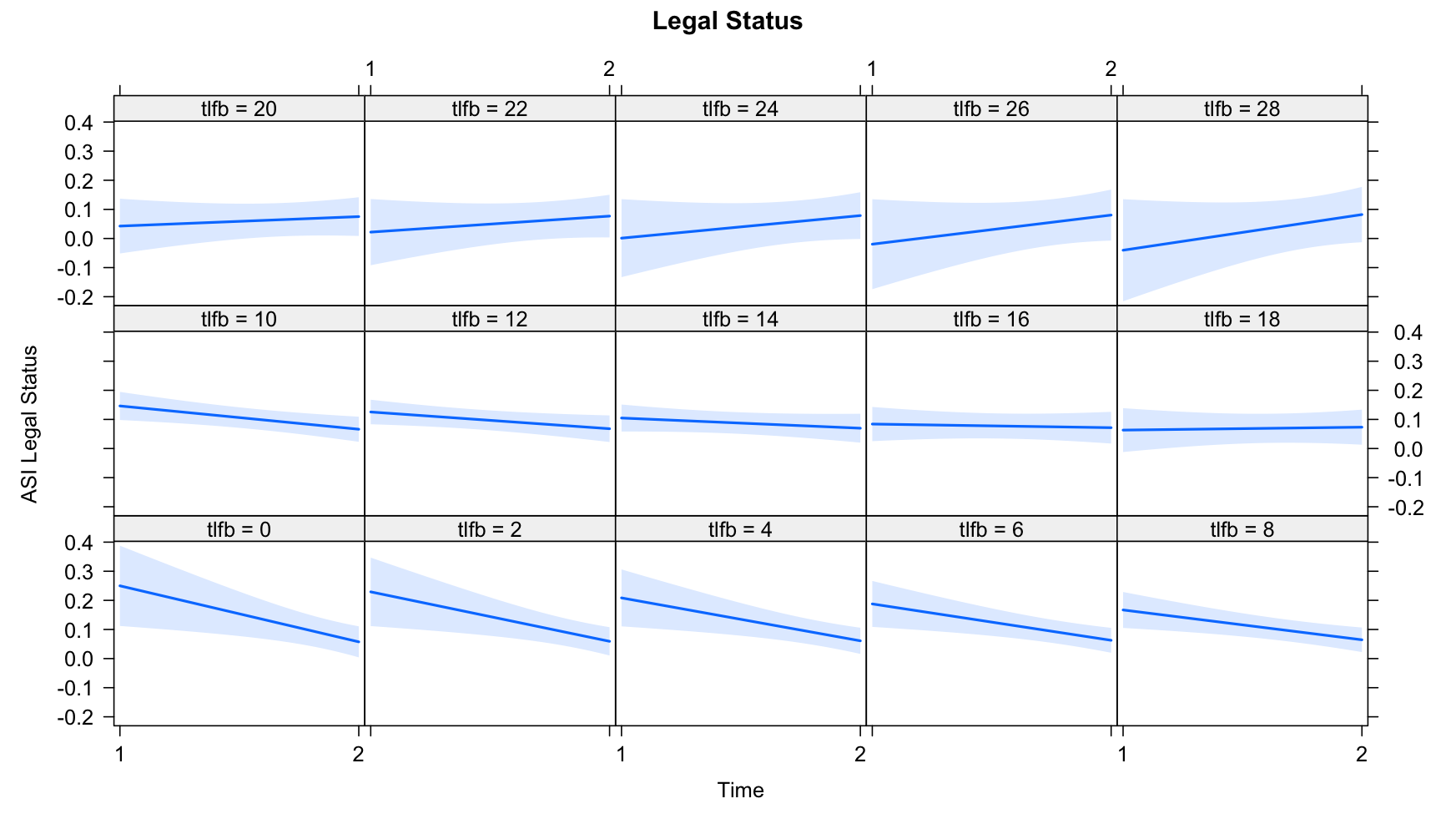** |

**Supplemental Figure 1. Effect plots of potential life problems over time for given opioid consumption frequency levels per 4-week period.**

Effect plots were derived from the generalized linear mixed model. Opioid consumption frequency levels during 28 days were reported as follows: no use (tlfb=0), 2 days of use (tlfb=2), …, 26 days of use (tlfb=26), use every day (tlfb=28). Opioid use was self-reported every two weeks using the Timeline Follow-Back (TLFB) questionnaire. Time 1 indicates measurements at baseline and time 2 indicates measurements at the end of the study (week 24). ASI=Addiction severity index; TLFB= Timeline Follow-Back.
